# Design of a Novel Sericite–Phosphoric Acid Framework for Enhancement of Pb(II) Adsorption

**DOI:** 10.3390/molecules28217395

**Published:** 2023-11-02

**Authors:** Han-Soo Kim, Hee-Jeong Choi

**Affiliations:** Department of Biomedical Sciences, Catholic Kwandong University, Beomil-ro 579 beon-gil, Gangneung-si 25601, Republic of Korea; hansk@cku.ac.kr

**Keywords:** adsorption, clay, kinetic, lead removal, sericite bead

## Abstract

In this study, phosphoric acid was used to attach anions to the weak interlayer structure of sericite, one of the clay minerals composed of a tetrahedral structure of silicate, to increase the adsorption capacity of cations. Natural sericite beads (NSB) and activated sericite beads with phosphoric acid (PSB) were prepared as beads in order to increase reusability and facilitate the separation of adsorbates and adsorbents. Using this, lead (Pb(II)) removal efficiency from an aqueous solution was comparatively analyzed. The pHpzc was 6.43 in NSB but lowered to 3.96 in PSB, confirming that more acidic functional groups were attached to the PSB surface. According to FT-IR analysis, P=O, P-O-C, P=OOH and P-O-P bonds appeared on the surface of the PSB adsorbent, and the peaks of carboxyl groups and OH-groups were large and broad. The maximum adsorption capacity of Langmuir was 52.08 mg/g for NSB and 163.93 mg/g for PSB. The adsorption process was close to physical adsorption for NSB and chemical adsorption for PSB, and both adsorbents were endothermic reactions in nature in that the higher the temperature, the higher the adsorption efficiency. The adsorption mechanism of Pb(II) to PSB was achieved by ion exchange, electrostatic interaction, hydrogen bonding, and complexation. The adsorption of Pb(II) using PSB was not significantly affected by the adsorption of competing ions and showed a high adsorption efficiency of 94% in reuse up to 6 times. This confirms the favorable feasibility of removing Pb(II) from industrial wastewater using PSB.

## 1. Introduction

In recent years, biomass or clay minerals, which boast abundant materials readily available everywhere, have attracted more and more attention due to their low cost, eco-friendliness, and reusability [1,2]. However, most natural adsorbents have low adsorption efficiency [3,4], and, in some cases, low adsorbent strength and low selectivity for heavy metals to be adsorbed in the aqueous solution [5,6]. In order to overcome these shortcomings, the researchers reported that the adsorbent was chemically modified through various techniques, thereby obtaining an adsorbent with improved adsorption efficiency and adsorption strength [7,8,9]. Sericite, which is abundant in nature and inexpensive, is a very attractive heavy metal adsorbent.

The main components of sericite are SiO_2_ (45.2%), Al_2_O_3_ (38.5%), K_2_O (11.8%), and H_2_O (4.5%) [1,10], and it is composed of a tetrahedral structure of silicate [2,11]. These tetrahedrons (tetrahedron: T-layer) are connected to each other, creating a single face in the front and rear left and right [6,10]. Layer T faces each other upside down, with octahedral sites interposed between them [2]. This site is called the O-layer. Thus, the T-layer, the O-layer, and the T-layer again meet to form a single layer, which is spread out in two dimensions. This layer is called the T-O-T layer. Hydrogen often attaches to oxygen exposure on the surface of the T-O-T layer to form a hydroxyl group (-OH group), and a cation enters between the T-O-T layer and another T-O-T layer [12]. It contains a monovalent cation, and the bond is a van der Waals bond. This cation becomes the atom that connects each layer.

In the tetrahedral site (T-layer) of sericite, Al substitutes a lot of Si, and the ratio is Al:Si = 1:3 [11]. When divalent cations fill the octahedral sites, all the vacancies are filled, and, at this time, one oxygen comes into contact with three cations [10]. This is called trioctahedral. On the other hand, when trivalent cations fill the octahedral sites, vacancies should be formed little by little on a regular basis [6,12]. In this case, oxygen meets two positive ions. Therefore, it is called a dioctahedral. The mica belonging to the double octahedral type is sericite. After such arrangement, each T-O-T unit is in a monovalent anion state [2,13]. That is, more monovalent cations are needed. The input at this time is K^+^. This is the cation that connects the T-O-T layer and the T-O-T layer. It can be easily assumed that some of the K components are substituted by other metals. In this very structure, the attractive force between T-O-T and T-O-T is very weak. According to previous studies [11,13], the presence of hydroxyl groups on the surface of natural sericite can easily remove various contaminants in water such as dyes and heavy metal ions. However, sericite does not swell in water and has low cation exchange capacity compared to other clays such as montmorillonite and bentonite due to potassium ions strongly bound to the interlayer surface [5,14]. Therefore, in order to convert natural sericite into a more valuable adsorbent by improving its surface area, porosity, and sorption capacity, it needs to undergo an activation process [6,10,11,12]. Thus, this study attempted to increase the adsorption capacity of cations by attaching anions to this weak structure using phosphoric acid.

The acid activation of sericite can be mainly activated using phosphoric acid and sulfonic acid; however, when the adsorbent is activated using phosphoric acid, there are many advantages of efficiently adsorbing heavy metals in aqueous solution. First, the surface area and porosity of the adsorbent are increased, and various functional groups such as -C=O, -C-O-C-, -OH, -P=O, -P-O-C and P=OOH can be attached to the adsorbent surface [15,16]. In particular, phosphorus-containing groups attached to the adsorbent during phosphorylation on the one hand promote a bond cleavage reaction. On the other hand, it promotes crosslinking through cyclization and condensation to form a bonding layer such as phosphate and polyphosphate ester that can protect the internal pore structure, acting as an excellent heavy metal adsorbent in an aqueous solution [17]. According to previous studies [8,16,18], phosphoric acid-activated adsorbents can be recycled and have various advantages such as low toxicity. So far, many studies have been published on the adsorption of various heavy metals and dyes using sericite [6,10,11,12,13,19,20], but there are very few papers on the phosphorylation of sericite to remove Pb(II) from an aqueous solution and analyze the removal efficiency.

Therefore, in this study, in order to increase the removal efficiency of Pb(II), which is one of the heavy metals emitted in the industrial process, sericite was modified using phosphoric acid, and it was manufactured into beads for easy adsorption and separation. In order to optimize the Pb(II) removal efficiency in an aqueous solution, characterization of the adsorbent and various parameter experiments were conducted. Using these, adsorption kinetics, adsorption isothermal, and adsorption mechanism were analyzed, and thermodynamic analysis was performed using Gibb’s free energy. Finally, natural sericite beads (NSB) and activated sericite beads with phosphoric acid (PSB) reuse experiments were conducted to evaluate the economic feasibility of the adsorbent.

## 2. Results and Discussion

### 2.1. Characteristics of Adsorbents

#### 2.1.1. Physical Characteristics

The main components of NSB and PSB were oxygen and Si, and in addition, Al, K, and a small amount of carbon were contained (Table 1). After the phosphorylation of NSB, the oxygen content increased and the carbon content decreased. This may be due to the release of volatiles (C-H aromatic) during the phosphorylation process, and the relatively high oxygen content is thought to be due to the high level of chemical actives (H_3_PO_4_) used. In addition, the P content of PSB increased, and it was confirmed that NSB was successfully phosphorylated. After phosphorylation of NSB, the ratio of O/C increased about 32 fold from 36.04 to 1164.40. It can be seen that the carboxyl groups inside the adsorbent increased after phosphorylation of NSB, and in particular, the oxygen content increased after phosphorylation, and thus the number of groups containing oxygen in the adsorbent increased. According to previous studies [21,22], the higher the O/C ratio, the greater the number of carboxylate groups on the adsorbent surface. Due to this, the anionic properties of the adsorbent are strengthened and the adsorption capacity of the cationic material is increased.

The pore size of the adsorbent is also a major influencing factor affecting the adsorption efficiency [22]. According to the previous study [6,10,11,21,22], compared to ultra-micropore (less than 1.0 nm) and macropore size (more than 50 nm), the adsorption efficiency of Pb(II) was about 3 times higher in micro-meso (2–50 nm) size. In NSB, 89.2% of micropores of <2 nm and 10.3% of mesopores were particles with micropores rather than mesopores. However, after phosphorylation of NSB, 97.2% fell within the mesopore range, confirming that the overall pore size of the adsorbent increased. In addition, the BET surface area increased markedly with the increase in the adsorbent in the mesopore range, which is thought to affect the adsorption efficiency of Pb(II) in aqueous solution along with the change in pore size.

#### 2.1.2. Surface Morphology and FTIR Spectra

**Figure 1:**
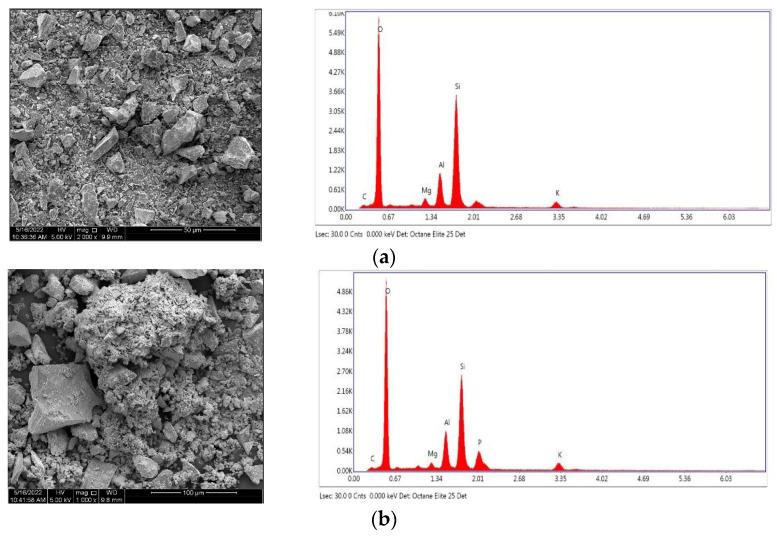
A SEM image and EDS of (**a**) NSB (**b**) PSB.

The surface morphology and the major components of NSB and PSB were observed by SEM and EDS, and the results are shown in Figure 1. According to SEM observation, the surface of NSB, which was smooth, became rough after phosphorylation, and the pores were enlarged. This change in adsorbent surface provides a great advantage in the adsorption of harmful substances in an aqueous solution because it can provide more adsorption sites than a smooth adsorbent surface. As a result of EDS analysis, it was confirmed that PSB contained phosphoric acid.

**Figure 2:**
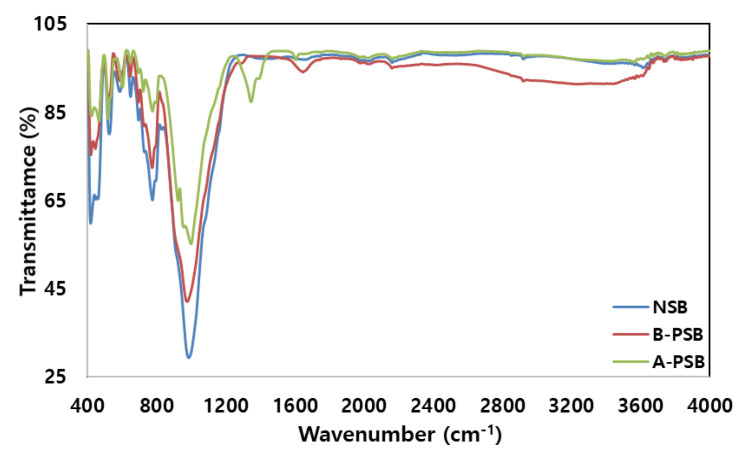
FTIR spectra of NSB, PSB before (B-PSB) and after (A-PSB) adsorption of Pb(II).

The FTIR spectrum of the adsorbent showed band broadening, enhancement, and reduction of peaks in certain areas after phosphorylation; however, the basic FTIR patterns of NSB and PSB were similar (Figure 2). The basic FTIR patterns of NSB and PSB were N-containing bioligands (400–425 cm^−1^) [10], O-H aromatic and Si-O of quartz and silica (625–830 cm^−1^), and C-H aromatic or PO_4_^3−^ stretching (890–1160 cm^−1^). In particular, strong vibration was observed in the range of 400–1300 cm^−1^. After modification of the NSB with phosphoric acid, three major changes were detected in the FTIR peak. Firstly, the width of the peaks of N-containing bioligands (400–425 cm^−1^), O-H aromatic and Si-O of quartz and silica (625–830 cm^−1^), and C-H aromatic or PO_4_^3−^ stretching (900–1160 cm^−1^) hardly changed, but the depth decreased. Secondly, the C=O peak (1725–1750 cm^−1^) in aldehydes, ketones, and carboxyl acids groups were newly formed. Thirdly, a very broad typical hydrogen-bonded hydroxyl group (-OH) was formed in a range from 2800 to 3600 cm^−1^. The reasons for these three main phenomena are that the oxygen contained in NSB combined with the phosphorus–carbon compound (hydrogen bonding P=O groups, P-O-C (aromatic) bonds, and P=OOH of phosphate or polyphosphate, etc.) to form a new group due to phosphorylation of NSB. In particular, the decrease in the 900–1160 cm^−1^ peak was due to the phosphorylation of NSB, and the symmetrical part of P+O- of the phosphoric acid ester and P-O-P of the polyphosphoric acid chain was combined with C-H aromatic to form a C=O carboxylic acid (1725–1750 cm^−1^) and a hydroxyl group (2800 to 3600 cm^−1^). Carboxylic acids can be attached to two or more atoms or groups of atoms and become polar due to charge separation between oxygen and carbon atoms [21]. In an aqueous solution, the O-H bond of the carboxyl group is easily broken to form O- and H^+^ [22,23]. Therefore, the carboxyl group in aqueous solution plays an important role in adsorbing positive heavy metals such as Pb(II). The surface of the phosphoric acid-modified adsorbent contained significant amounts of phosphorus compounds, and the surface area and porosity of the adsorbent increased due to the phosphorylation of NSB. In conclusion, the presence of functional groups such as -C=O, -COC-, -OH, -P=O, -P-O-C and P=OOH on the surface of the adsorbent is expected to play a role in increasing the adsorption capacity of Pb(II) in aqueous solution.

#### 2.1.3. Point of Zero Charge (pzc)

**Figure 3:**
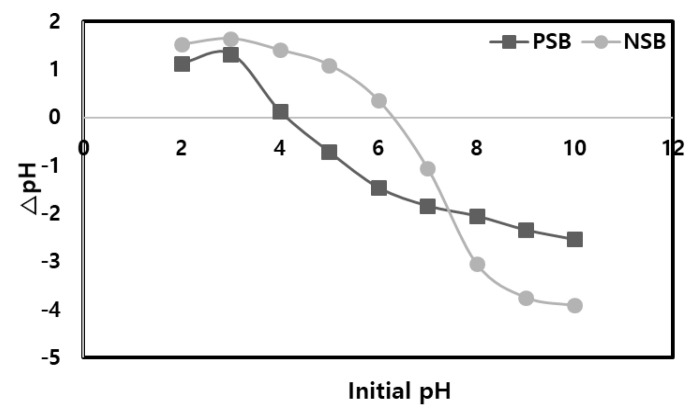
The pHpzc for NSB and PSB in aqueous solution.

The efficiency of a specific adsorption process relies on the pHpzc (point of zero charge) of the biosorbent and the specific metal ion it is targeting [10,24]. pHpzc is the pH (of the solution) value at which the surface charge of the biosorbent becomes zero [21]. Beyond the isoelectric point, when the pH is higher, the adsorbent’s surface adopts a negative charge, leading to a robust electrostatic interaction with positively charged adsorbent materials, resulting in increased adsorption efficiency [21,23]. Proton bonding on the majority of carbon materials displays both positive aspects, involving proton adsorption, and negative aspects, involving proton emission [16,25]. The addition of phosphoric acid to NSB leads to the creation of a carbon surface with acidic properties, resulting in a rapid decrease in the pzc (point of zero charge) from its initial neutral value of 6.37 to the acidic range of 3.92, as depicted in Figure 3. This observation indicates a strong attachment of acidic groups to the surface of PSB. In addition, it means that the pH range at which Pb(II) ions can be adsorbed in an aqueous solution is widened, which is thought to Pb(II) to increase in adsorption capacity.

### 2.2. Parametric Study

#### 2.2.1. Dose of Adsorbent

**Figure 4:**
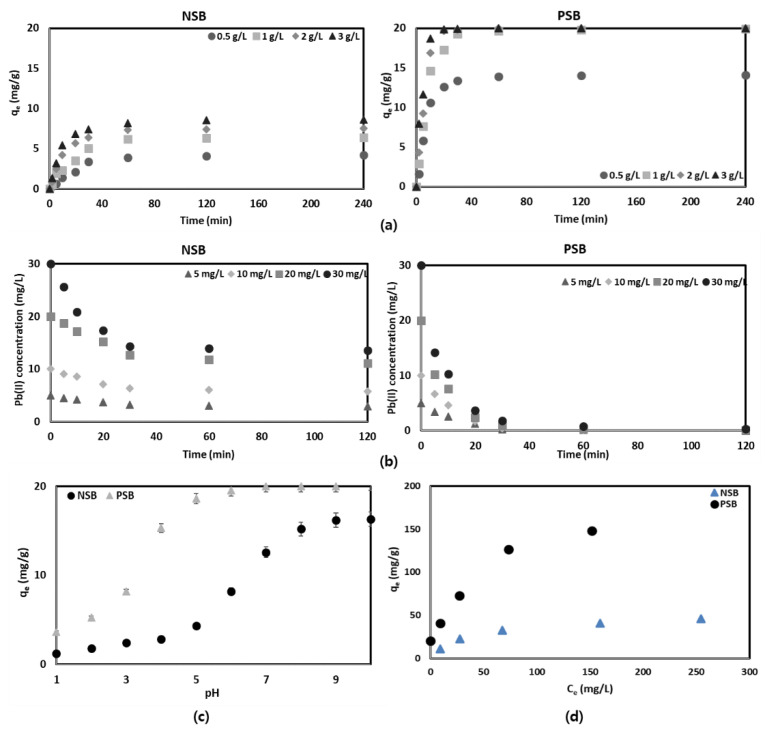
Effect of (**a**) various doses; (**b**) various initial concentrations; (**c**) various pH; (**d**) qe vs. Ce of NSB and PSB for removal of Pb(II).

The effect of the amount of NSB and PSB on the adsorption efficiency of Pb(II) in aqueous solution was tested in a range of 0.5–3.0 g/L, and the results were calculated (Figure 4a). Being able to adsorb a large amount of harmful substances using a small amount of adsorbent is a very favorable signal from both economic and environmental aspects. As can be seen in Figure 4a, it can be confirmed that PSB has about 2.32–3.35 times higher adsorption efficiency than NSB, and reached the maximum adsorption amount rapidly. This result is thought to be because, as already mentioned in the physical analysis of the adsorbent and the surface analysis of the adsorbent, the pore size of the adsorbent was changed in favor of Pb(II) adsorption after the introduction and phosphorylation of various functional groups and phosphate groups on the surface of the adsorbent. This is in agreement with many previous research results [2,6,10,11,23,25].

In the case of using PSB, there was a difference in the adsorption amount within 20 min of the test, but 20 mg/g of Pb(II) was adsorbed within 30 min on all adsorbents except for the 0.5 g adsorbent. In general, as the amount of adsorbent increases, the adsorption capacity increases because the number of active sites capable of adsorbing the adsorbent increases. However, according to the results of several previous experiments, it was observed that, after reaching the maximum adsorption efficiency, the adsorption capacity did not increase or even started to decrease even if the amount of adsorbent was increased [7,16,21,22,26]. The reason is that if too much adsorbent is administered, the adsorbent particles start to agglomerate, and the active sites start to overlap each other, thereby blocking a mass transfer. In order to solve these problems, increasing the stirring speed of the aqueous solution helps to overcome the mass transfer resistance, but it must also be maintained within a certain range. This is because if the stirring speed is too high, fragmentation of the adsorbent may occur. Therefore, according to the experimental results, 1 g/L of PSB is recommended for the adsorption of Pb(II) in aqueous solution.

#### 2.2.2. Initial Concentration

The effect of the initial concentration of Pb(II) in the aqueous solution on the adsorption was tested using the amounts of NSB and PSB of 1 g/L in the range of Pb(II) concentration of 0–30 mg/L. This is because, in general, the concentration of Pb(II) does not exceed 20 mg/L in industrial wastewater except in special cases. In addition, it is because the adsorption amount of 1 g/L was excellent in the experiment on the effect of the above-mentioned amount of adsorbent on adsorption. As can be seen in Figure 4b, the Pb(II) adsorption process using NSB and PSB in an aqueous solution was significantly different. In the case of NSB, the adsorption rate was significantly slower than that of PSB, and the adsorption efficiency was almost similar regardless of the concentration. This is because the adsorption sites that can adsorb Pb(II) to the adsorbent are limited, and the number of functional groups on the adsorbent surface that attract the Pb(II) are smaller than those of PSB. The adsorption efficiency of Pb(II) onto NSB reached a range of 42.0–55.2% in 120 min, whereas PSB was found to be over 95% within 30 min. This confirms that PSB modified with sericite with phosphoric acid is very effective as an adsorbent capable of removing Pb(II) from an aqueous solution.

#### 2.2.3. Effect of pH

Functional groups on the surface of the adsorbent may vary depending on the pH of the aqueous solution [22]. Depending on the pH of the aqueous solution, pH < pKa_1_ (2.15), pKa_1_ < pH < pKa_2_ (6.8), and pH > pKa_2_, the phosphoric acid functional group on the adsorbent surface can be changed in the form of R-PO_3_H_2_, R-PO_3_H^−^, and R-PO_3_^2−^, respectively, which may affect the adsorption of Pb(II) in aqueous solution [21,27]. It was confirmed that the adsorption of Pb(II) in aqueous solution increased rapidly from pH 7 and PSB above pH 4 when NSB was used (Figure 4c). This phenomenon is related to the above-mentioned value of pHpzc. This is because, when the pH is higher than pHpzc, the surface of the adsorbent becomes negatively charged, making it easier to adsorb cationic heavy metals [22,28]. Almost all heavy metal ions, including Pb(II), exist as cations in aqueous solutions. Therefore, a sharp increase in the adsorption efficiency was observed when the pH value exceeded the pHpzc value (pH > pHpzc). Also, as the pH increases, the carboxyl and phenolic hydroxyl groups of the PSB adsorbent are deprotonated to form -P=O, -P-O-C, P=OOH, R-COO- and R-O- groups [22,28,29]. Due to this, the surface of the PSB adsorbent has a strong negative charge, and the electrostatic attraction between the PSB adsorbent and the cationic ion of the aqueous solution, Pb(II), is improved so that the adsorption efficiency of Pb(II) is increased.

### 2.3. Adsorption Kinetics and Isotherms

#### 2.3.1. Adsorption Kinetics

**Figure 5:**
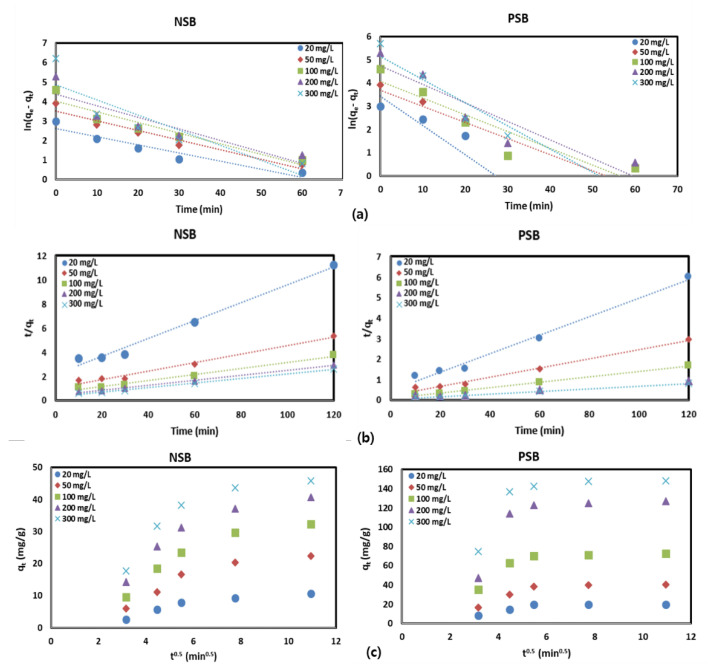
A linear plot of (**a**) PFO, (**b**) PSO, and (**c**) ID for adsorption of Pb(II) on NSB and PSB.

Analysis of adsorption kinetics can provide information on the physicochemical interactions, mass transport, and adsorption rates between adsorbents and adsorbate during the adsorption process [4,8,9]. This helps to understand the mechanism and kinetics of the adsorption of Pb(II) on NSB and PSB in aqueous solution and to determine the optimal conditions for adsorption. For the analysis of adsorption kinetics, pseudo-first-order (PFO), pseudo-second-order (PSO), and intraparticle diffusion (ID) models were used. The experimental results on adsorption kinetics were analyzed and the results are presented in Figure 5 and summarized in Table 2.

As a result of the analysis of adsorption kinetics, q_e,cal_ values calculated using the PSO model were closer to q_e,exp_ than for both NSB and PSB. The correlation coefficient (R^2^) was higher in PSO than in PFO, and χ^2^ was lower in PSO than in PFO. Therefore, the adsorption of Pb(II) onto NSB and PSB was more suitable for PFO than for PSO. Moreover, the values of k_1_ and k_2_ of PFO and PSO decreased as the concentration of Pb(II) increased in both NSB and PSB adsorbents. This indicates that the higher the concentration of Pb(II) in the aqueous solution, the lower the active sites that can be adsorbed to the adsorbent. The analysis of the adsorption process using the ID model informs the diffusion rate of the adsorbent into the adsorbent. The plot of q_t_ versus t_0.5_ for Pb(II) adsorption with NSB and PSB (Figure 5c) did not pass through the origin, so intraparticle diffusion is not the only rate-limiting step. According to Figure 5c, it can be seen that pore diffusion and intraparticle diffusion are the rate-limiting steps in the Pb(II) adsorption process using NSB and PSB. The values of k_id_ and C calculated from the slopes of the plots increased as the Pb(II) concentration of the aqueous solution increased. In particular, the larger the value of C representing the thickness of the boundary layer, the greater the contribution of surface sorption in the speed control step [21,30]. It is thought that the sorption contribution of the adsorbent surface to the adsorbent material has a direct effect on the adsorption efficiency.

#### 2.3.2. Adsorption Isotherm

**Figure 6:**
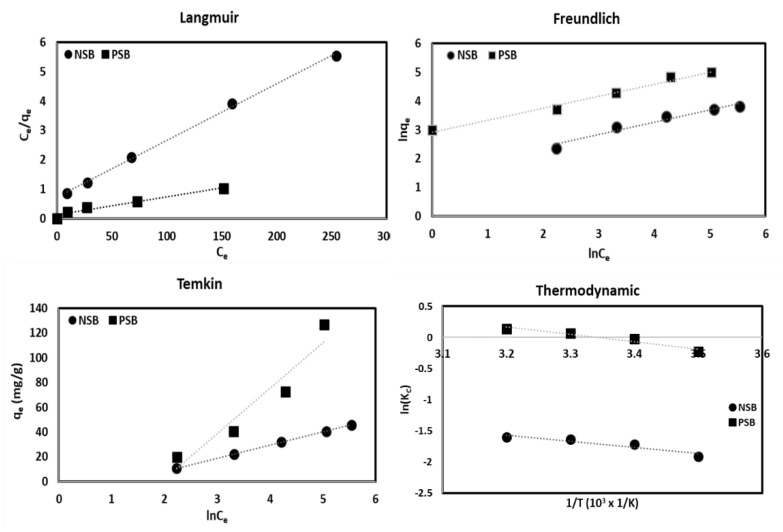
A plot of Langmuir, Freundlich, Temkin and Thermodynamic of Pb(II) onto NSB and PSB.

The interpretation of the adsorption isotherm helps to understand the adsorption mechanism and helps to determine the arrangement of adsorption sites and the adsorption of metal ions on the adsorbent surface [10,17,22,28]. The results of the isothermal adsorption experiment were analyzed using the models of Langmuir, Freundlich, and Temkin and summarized in Table 3 and Figure 6.

Dimensionless separation factor (R_L_ (=1/(1 + K_L_Co)) calculated in the Langmuir equation was found to be 0.1141 for NSB and 0.0631 for PSB, making the adsorption process favorable. Temkin isothermal adsorption constant (B) was calculated at 10.60 for NSB and 36.79 (J/mol) for PSB, NSB corresponds to the physical adsorption region (B < 20 J/mol), whereas PSB corresponds to the chemical adsorption region (B > 20 J/mol). Therefore, the adsorption of Pb(II) by NSB was closer to physical adsorption caused by the action of the van der Waals force, which consists of dispersing and electrostatic forces, rather than chemical adsorption, in which the chemical form of the adsorbent changes due to the reaction between the adsorbent and the adsorbent. However, adsorption by PSB was closer to chemical adsorption by hydrogen bonding, ion exchange, covalent bond, ionic bond, and complexation due to various functional groups on the adsorbent surfaces and Pb(II). The K_F_, which represents a measure of adsorption capacity calculated from Freundlich’s equation, indicates that the higher the value, the better the adsorption capacity [22,31]. K_F_ was found to be 35.30 for NSB and 834.45 for PSB, which is about 23 times larger than that of NSB, indicating that PSB has superior adsorption capacity than NSB. To be sure, 1/n, which represents the adsorption strength, was 2.33 and 2.39 for NSB and PSB, respectively, and the adsorption strength of Pb(II) for NSB and PSB in aqueous solution was in the strong range (0–1).

The correlation coefficient (R^2^), which shows the applicability of various adsorption models, was higher in the Langmuir model than that of Freundlich or Temkin for both NSB and PSB. Therefore, it is judged that the adsorption of Pb(II) to NSB and PSB is most suitable for the Langmuir model. However, the correlation coefficient for the Freundlich and Temkin models is also high, so the adsorption of Pb(II) on NSB and PSB can be applied to the Freundlich model as well. The Langmuir maximum adsorption amount was 52.08 for NSB and 163.93 mg/g for PSB. This is related to the complex adsorption mechanism due to the various functional groups on the adsorbent surface mentioned above.

### 2.4. Thermodynamic Interpretation

Thermodynamic analysis was performed using Gibb’s free energy, and the results are presented in Table 4 and Figure 6. In the adsorption process, the temperature of the aqueous solution can affect the solubility of metal ions and potential ion exchange materials [21,32]. This can have a decisive effect on the adsorption rate and adsorption efficiency, since the solubility of metal ions may induce ion exchange on the adsorbent surface. The slope and intercept of lnk_c_ vs. 1/T in Figure 6 were used to calculate the values of ΔH and ΔS. The value of ΔG was negative for both NSB and PSB (Table 4), so the adsorption process was spontaneous. Since ΔH and ΔS are positive values (Table 4), the adsorption process of Pb(II) in aqueous solution using NSB and PSB is an endothermic reaction in which the adsorption efficiency increases as the temperature increases, and the randomness of the adsorbent surface increases. It is thought that as the temperature increases, the kinetic energy of the molecules in the aqueous solution increases, so the molecular motion becomes active and entropy increases [11,33]. Opinions on exothermic and endothermic reactions in previous studies on the adsorption process of hazardous substances in aqueous solutions differ depending on the type of adsorbent. This is because the stability of the ligand and the adsorbent and the solubility of the adsorbent depends on the temperature. According to previous studies [7,16,18,20,21,30], most of the adsorption processes were observed as endothermic reactions. This is because, when the temperature of the aqueous solution increases, the surface activity of the adsorbent increases, and the thickness of the boundary layer surrounding the adsorbent decreases, thereby increasing the removal efficiency.

### 2.5. Adsorption Mechanism

The higher the O/C ratio, the anionic properties of the adsorbent are strengthened and the adsorption capacity of the cationic material is increased. After phosphorylation of NSB, the ratio of O/C increased about 32 fold from 36.04 to 1164.40. It can be seen that the oxygen content increased after phosphorylation, and thus the number of functional groups containing oxygen in the adsorbent increased. In addition, the range of mesopores and the BET surface area of PSB were found to increase significantly after phosphorylation of NSB, which was a factor in improving the adsorption efficiency of Pb(II) in aqueous solution.

There are many functional groups that can absorb metal cations reported in the literature, and the nature of these functional groups was highly pH dependent [16,22]. PSB had a lower pHpzc than NSB, so the pH range for cation adsorption was wide. In addition, PSB, a strong acid resin, has an ion exchange capacity due to the phosphoric acid group contained in the adsorbent. Strong acid resins adsorb almost all the cations contained in the aqueous solution by exchanging them with hydrogen ions [22,27]. Since this ion exchange process is reversible, it has the advantage that the resins can be regenerated using strong acids when the ion exchange capacity is exhausted [21,30].

**Figure 7:**
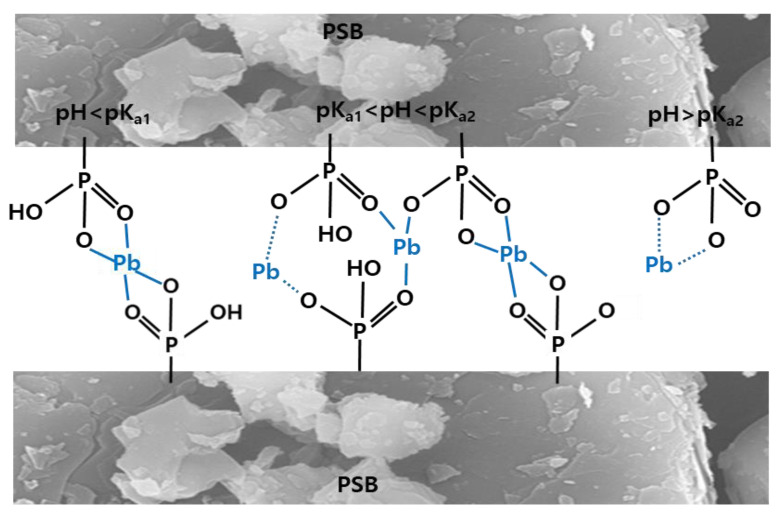
Possible mechanism of Pb(II) sorption for PSB-bearing phosphoric acid moieties.

A possible Pb(II) adsorption mechanism of sericite with phosphoric acid in aqueous solution was explained in Figure 7. NSB adsorbed Pb(II) in an aqueous solution by electrostatic interactions and ion exchange. However, PSB is adsorbed through ion exchange, electrostatic interaction, hydrogen bonding, and complexation depending on the relationship between pH and pKa. In addition, since all PSBs were reformed with strong acids, Pb(II) removal by precipitation is also possible.

The surface functional groups of the adsorbent play an important role in bonding the adsorbent surface to the adsorbate. NSB is suitable for adsorbing positively charged contaminants because their surface is negatively charged. However, the negative charge on the surface of NSB is weak and the functional groups are not diverse, so the removal efficiency of positively charged heavy metals is low. PSB strengthened the adsorption efficiency of cations by attaching anions to the weak structure of the T-O-T layer of NSB by phosphorylation. FTIR analysis showed that phosphorylation and sulfonation of sericite improved the number of carboxyl groups (-COOH), and increased porosity and carbon surface area. As a result, the adsorption efficiency of Pb(II), which is a cationic ion, for PSB was improved. The process of adsorbing Pb(II) onto PSB in aqueous solution can be summarized as follows.

R-OH + Pb(NO_3_)_2_ → R-O-Pb + H(NO_3_)_2_ (1)

R-NH_2_ + Pb(NO_3_)_2_ → R-N-Pb + H_2_(NO_3_)_2_ (2)

R-COOH + Pb(NO_3_)_2_ → R-COO-Pb + H(NO_3_)_2_ (3)

R-POH + Pb(NO_3_)_2_ → R-PO-Pb + H(NO_3_)_2_ (4)

R-PO_3_H + Pb(NO_3_)_2_ → R-PO_4_-Pb + H(NO_3_)_2_ (5)

Combining the above experimental results, the oxygen-containing functional group content of the modified PSB with a larger surface area was greater than that of NSB, and PSB adsorbed Pb(II)) much more strongly than NSB. In addition, the surface area of PSB modified with phosphoric acid had a significant effect on the sorption of Pb(II). PSB adsorbed about 4.6 times more Pb(II) than NSB. This is because groups such as P=O and P=OOH attached to the surface of PSB interacted with Pb(II) ions and formed a complex in aqueous solution, and in FT-IR analysis, the amount of carboxyl (-COOH) and hydroxyl (-OH) functional groups of PSB was higher than that of NSB.

### 2.6. Interference of Other Ions

In actual industrial wastewater, various heavy metals are mixed. However, most of the studies focused on single heavy metals do not consider the effects of interference and co-precipitation of other ions in the study of the adsorption properties of heavy metal ions of adsorbent materials. This indicates a limitation in reflecting the competition between heavy metal ions in actual industrial wastewater or the adsorption characteristics of mixed heavy metals. Therefore, 20 mg/L of each of the various competing ions (Cd^2+^, Ni^2+^, Cu^2+^, Zn^2+^, Fe^3+^, Cr^6+^), which are most commonly present in industrial wastewater, were mixed, and the adsorption efficiency was comparatively analyzed.

**Figure 8:**
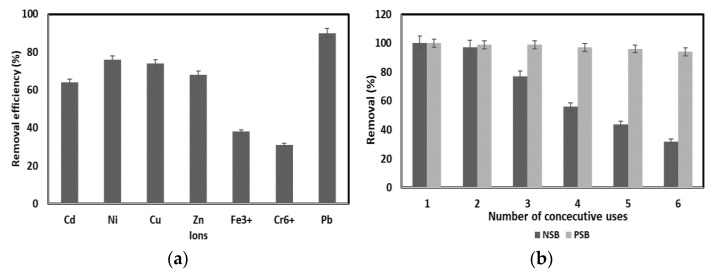
(**a**) Effect of competing ions. (**b**) Reuse of NSB and PSB for Pb(II) removal in aqueous solution.

As a result of the experiment, monovalent and divalent cations (Cd, Ni, Cu, Zn) showed 64–76% removal efficiency, which was higher than the 31–38% removal efficiency of trivalent and hexavalent heavy metals Fe and Cr (Figure 8a). Although the adsorption efficiency of Pb(II) decreased compared to the single ion experiment without competing ions, the adsorption efficiency was 88–92%, indicating that it was not significantly affected by other ions. This is a favorable signal that there is a possibility of effectively adsorbing Pb(II) by applying PSB to actual industrial wastewater.

### 2.7. Reusability

In general, the adsorbent is reused after washing with HCl, NaOH or water, but in this experiment, washing with acids and bases was not performed [9,16,17,18,33]. This is to reduce the cost and environmental pollution caused by the treatment of the adsorbent washing material and to secure excellent economic efficiency through the reuse of the adsorbent. Most industrial wastewater contains Pb(II) in a concentration of less than 20 mg/L. Thus, the concentration of Pb(II) was set to 20 mg/L for the test. After the first experiment, the adsorbent was dried in an oven at 75 °C for 5 h, and then the second experiment was performed under the same conditions as the first experiment.

When PSB was reused to adsorb Pb(II), it showed a high adsorption efficiency of 94% even after reusing 6 times to adsorb Pb(II), whereas NSB showed an adsorption efficiency of 97% until reused twice. In addition, the adsorption efficiency of NSB decreased sharply from the third reuse, and the fifth reuse showed an adsorption efficiency of 44%, showing an adsorption efficiency of less than 50% (Figure 8b). Therefore, as mentioned above, PSB can be applied as a Pb(II) adsorbent in industrial wastewater because of its strong adsorption strength, reusability of the adsorbent, and excellent adsorption efficiency.

## 3. Materials and Methods

### 3.1. Materials

#### 3.1.1. Phosphorylation of Sericite

Sericite having a particle size of less than 100 mesh (0.254 mm) was collected and washed with distilled water. After removing contaminants, sericite was dried at 75 ± 2 °C for 48 h to evaporate moisture. The method of activating dried sericite using phosphoric acid (H_3_PO_4_) as a porous adsorbent is as follows. First, 200 mL of phosphoric acid (85%) and 75 g of phosphorus pentoxide were mixed and completely dissolved while heating and stirring and then cooled at room temperature. The ratio of prepared phosphoric acid solution and distilled water was 10:1 to prepare 1000 mL of a mixed solution. Next, 20 g of sericite was added to this mixed solution, and the sericite was activated while mixing at 120 rpm at 35 ± 2 °C for 5 h. After that, it was left at room temperature for 4 h to allow the reaction to occur completely, and then 1N sodium hydroxide was added to adjust the pH of the adsorbent to be in the range of 7.0 to 7.5. Then, the adsorbent was sufficiently washed with distilled water, and it was put in a dryer and dried for 48 h. The adsorbent in the form of fine powder is not easily separated from the aqueous solution after the adsorption process, and there is a pressure loss in the fixed bed column, so it is difficult to apply in the field. Therefore, in this study, the adsorbents were prepared in the form of beads that are easy to separate after the adsorption process. Sericite and phosphoric acid-activated sericite were mixed with a little distilled water and kneaded under constant pressure for 2 h to enhance the viscosity of the clay mineral. With this dough, spherical beads with a diameter of 0.3 cm were made, and the dried beads were ceramized in an oven at 800 °C. Finally, natural sericite beads (NSB) and phosphoric acid-activated sericite beads (PSB) were stored in a desiccator for use in experiments.

#### 3.1.2. Adsorbate

We utilized analytical grade Pb(NO_3_)_2_ (from Duksan Pure Chem., Co. Ltd., Ansan-si, Republic of Korea) with a purity of at least 99% in our experiment. To begin, a stock solution of Pb(II) at a concentration of 1000 mg/L was prepared, and subsequent solutions at the desired concentrations were sequentially prepared by diluting the stock solution with distilled water. 

### 3.2. Experiment and Analytical Methods

#### 3.2.1. Characterization of Samples

The adsorbent’s carbon and oxygen contents were quantified using an automated ‘Vario EL’ element analyzer (PerkinElmer, Waltham, MA, USA). For a comprehensive analysis of the surface and chemical composition of the adsorbent, Scanning Electron Microscopy (SEM) and Energy-dispersive X-ray Spectroscopy (EDS) were employed, using the JSM-IT500 instrument by JEOL Ltd. in Tokyo, Japan. The functional groups attached to each adsorbent were identified through Fourier transform infrared spectroscopy (FTIR) conducted in a scan range of 400–4000 cm^−1^ using the Perkin Elmer FTIR 1760X (USA). The BET (Brunauer Emmett Teller) surface area was determined via a BET Surface Analyzer (Quantachrome Instruments version 11.03), involving the adsorption/desorption isotherm of N_2_ conducted at 77 K. To measure the quantity of adsorbent, an electronic balance (XP26, Mettler Toledo, Swiss, Greifensee, Switzerland) was utilized, and pH levels were measured using a pH meter (SevenGO pro, Mettler Toledo).

#### 3.2.2. Parametric Study

The experiment compared the adsorption efficiency of Pb(II) in aqueous solution using NSB and PSB and was carried out in the form of a batch test. The prepared adsorbent and Pb(II) solution were put into a 1 L Erlenmeyer flask according to the experimental conditions and sampled at a set time (0, 2, 5, 10, 20, 30, 60 and 120 min) while stirring at 120 rpm in a shaking incubator. Experiments on the effect of various parameters (adsorbent amount, pH, Temperature) on adsorption efficiency were conducted, and the amount of Pb(II) in each sample was determined using Atom Absorption Spectroscopy (AAS-6880 Shimadzu, Kyoto, Japan). From the measured values, the adsorption amount and adsorption efficiencies were calculated using Equations (6) and (7).

(6) $$q_{t}=\frac{(C_{0}-C_{t})V}{m}$$

(7) $$R=\frac{\left(C_{0}-C_{e}\right)}{C_{0}}\;\times \;100$$

where R is Pb(II) removal efficiency in aqueous solution.

Chi-square refers to the standard normal distribution and is a test to determine whether the difference between the expected frequency and the observed frequency is statistically significant. To select appropriate kinetic and isotherm models for the Pb(II) adsorption process, Chi-square (χ^2^) was calculated using Equation (8).

(8) $$\chi^{2}=\sum_{i=1}^{n}\;\frac{(q_{e,exp}-q_{e,cal}{)}^{2}}{q_{e,cal}}$$

where q_e,exp_ and q_e,cal_ are equilibrium adsorption capacities obtained from experiments and models, respectively.

#### 3.2.3. Reusability

To assess the adsorbent’s reusability, a resorption experiment was conducted eight times using the same adsorbent. The initial solution was prepared with 20 mg/L of Pb(II) solution and 1 g/L of adsorbent. The experiment was carried out at an aqueous solution temperature of 25 °C and pH 7. The initial solution was allowed to react with the adsorbent for 120 min, samples were taken, and the concentration of Pb(II) was measured using Atomic Absorption Spectroscopy (AAS). Removal efficiency was calculated using Equations (1) and (2). Following the first reuse test, the adsorbents were subjected to drying in an oven at 75 °C for 3 h. The second reuse experiment was performed under the same conditions as the first reuse experiment, utilizing the dried adsorbent. The process of reusing the adsorbent was repeated up to eight times, and notably, no desorption step using chemicals like NaOH or HCl was employed during this reusability assessment.

#### 3.2.4. Competing Ions

In the experiment investigating the influence of different heavy metal competitive ions on the removal efficiency of Pb(II), we selected Cr^6+^, Ni, Cd, Cu, Fe^3+^, and Zn, as these are commonly found in industrial wastewater discharges. Given that the concentration of Pb(II) typically present in on-site industrial wastewater is usually below 20 mg/L, we tested the concentration of the competing ions at 20 mg/L. The experimental conditions included 1 g/L of adsorbent, a pH of 7, and a room temperature of 25 °C.

#### 3.2.5. Adsorption Kinetic and Isotherm

The analysis of adsorption kinetics involved the application of various models, including the pseudo-first-order (PFO), pseudo-second-order (PSO), intra-particle diffusion (ID), as well as adsorption isotherms such as Langmuir, Freundlich, and Temkin. Additionally, thermodynamic analysis was conducted using the Gibbs free energy equation. Detailed mathematical equations and linearized forms of these adsorption kinetics and isothermal equations can be found in Table S1.

## 4. Conclusions

In this study, the anionic functional group was strengthened by the phosphorylation of sericite, and the removal efficiency of the cationic ion Pb(II) in aqueous solution was analyzed using this. After phosphorylation of NSB, the functional group of P=O groups, P-O-C (aromatic) bonds, P=OOH and P-O-P appeared on the surface of the PSB, and the carboxyl groups and OH-group peaks were large and broad. In addition, the specific surface area, mesopore range, and ion exchange capacity were significantly increased in PSB. The pHpzc of PSB was lower than that of NSB, confirming that an acidic functional group was attached to the PSB surface. The Langmuir maximum adsorption capacity was 52.08 and 163.93 mg/g for NSB and PSB, respectively, indicating that PSB showed about three times more adsorption than NSB. NSB was close to physical adsorption and PSB was close to chemical adsorption, and it was an endothermic reaction in which the adsorption efficiency increased as the temperature increased. In the reuse experiment, PSB showed a high adsorption efficiency of 94% up to 6 uses, and the adsorption of Pb(II) using PSB was not significantly affected by the adsorption of competing ions. The process of adsorption of Pb(II) to PSB was accomplished by ion exchange, electrostatic interaction, hydrogen bonding, and complexation. Also, since PSB was reformed with strong acid, Pb(II) removal by precipitation is possible. PSB has strong adsorption strength, can be reused as an adsorbent, and has excellent adsorption efficiency, so it can be applied as an adsorbent for Pb(II) in industrial wastewater.

## Figures and Tables

**Table 1:** Physico-chemical characteristics of before and after adsorption of Pb(II) for natural sericite bead (NSB) and phosphorylated sericite bead (PSB) (unit: atomic %).

Component		NSB	PSB
C		1.42	0.05
O		51.18	58.22
Al		7.65	7.87
Si		28.40	22.48
K		3.31	3.88
P		-	6.75
Porosity (%)		32.42 ± 5.26	81.97 ± 7.13
Pore size (nm)	<2	89.16 ± 4.85	0.32 ± 0.01
	2–50	10.26 ± 1.26	97.21 ± 5.48
	>50	0.54 ± 0.01	2.46 ± 0.03
BET surface area (m^2^/g)		2.34 ± 0.02	496.32 ± 6.75

**Table 2:** Kinetics parameters for the adsorption of Pb(II) onto NSB and PSB.

Parameters		Concentration (mg/L)				
		20	50	100	200	300
NSB						
q_e,exp_ (mg/g)		10.69	22.37	32.36	40.63	45.85
PFO	q_e,cal_	13.774	20.947	39.585	55.675	59.856
	k_1_	0.0417	0.0495	0.0545	0.0591	0.0779
	R^2^	0.9147	0.9381	0.9659	0.8178	0.79
	χ^2^	1.2849	1.3065	1.5263	1.9592	2.4654
PSO	q_e,cal_	13.459	21.5715	33.6826	47.3934	51.053
	k_2_	4.10 × 10^−4^	4.29 × 10^−5^	1.61 × 10^−5^	9.39 × 10^−6^	7.08 × 10^−6^
	R^2^	0.9839	0.9785	0.9887	0.9951	0.9936
	χ^2^	1.1032	1.1652	1.2631	1.4286	1.6549
ID	k_id_	0.9563	2.0148	2.7473	3.1034	3.2113
	C	1.1255	2.4606	5.2179	9.9824	14.956
	R^2^	0.8505	0.8474	0.8541	0.8339	0.7561
	χ^2^	1.3625	1.5895	1.8986	2.3265	2.7586
PSB						
q_e,exp_ (mg/g)		20.19	40.62	72.63	126.73	148.26
PFO	q_e,cal_	22.623	35.58	78.38	115.62	156.32
	k_1_	0.1261	0.0995	0.0798	0.0724	0.069
	R^2^	0.8575	0.9313	0.8553	0.8634	0.9467
	χ^2^	1.2016	1.2653	1.3265	1.5986	1.9872
PSO	q_e,cal_	21.027	42.045	76.519	128.889	152.25
	k_2_	9.36 × 10^−5^	1.09 × 10^−6^	2.15 × 10^−6^	3.73 × 10^−7^	2.62 × 10^−7^
	R^2^	0.9886	0.9893	0.9939	0.9795	0.9939
	χ^2^	1.0236	1.0862	1.1586	1.3265	1.5406
ID	k_id_	1.2615	2.5994	3.6962	7.4076	6.9651
	C	8.4918	16.525	38.859	60.065	85.61
	R^2^	0.5926	0.6191	0.5222	0.4528	0.4662
	χ^2^	1.145	1.2051	1.252	1.3851	1.8748

PFO: pseudo-first-order, PSO: pseudo-second-order, ID: intraparticle diffusion.

**Table 3:** Adsorption isotherms for Pb(II) on NSB and PSB at 298 K.

Isotherm	Parameters	NSB	PSB
Langmuir	q_m_	52.08	163.93
	K_L_	0.0259	0.0495
	R^2^	0.9982	0.9834
Freundlich	K_F_	35.30	834.45
	1/n	2.33	2.39
	R^2^	0.9552	0.9568
Temkin	B	10.60	36.79
	R^2^	0.9975	0.9153

**Table 4:** Thermodynamic parameters for adsorption of Pb(II) onto NSB and PSB.

Adsorbent	Temperature	Thermodynamic		
		ΔG° (KJ/mol)	ΔH° (J/mol)	ΔS° (J/mol·K)
NSB	288	−3.86	8.27	13.44
	298	−3.99		
	308	−4.13		
	318	−4.26		
PSB	288	−9.52	9.91	33.10
	298	−9.85		
	308	−10.18		
	318	−10.52		

## Data Availability

All data generated or analyzed during this study are included in this published article.

## Supplementary Materials

The following supporting information can be downloaded at: https://www.mdpi.com/article/10.3390/molecules28217395/s1, Table S1: adsorptin kinetic, isotherm, and thermodynamic models.
