# Supplementary material for: Design of a Novel Sericite–Phosphoric Acid Framework for Enhancement of Pb(II) Adsorption
Source: Molecules. 2023 Nov 2;28(21):7395. doi: 10.3390/molecules28217395 (PMC10649698; doi:10.3390/molecules28217395)
Supplement: Supplementary file 1 [file molecules-28-07395-s001.zip › molecules-2622043-supplementary.pdf]

Supplementary data

Table S1. Adsorptin kinetic, isotherm and thermodynamic models.

Model	Equation	Parameters
PFO	$\ln(q_e - q_t) = \ln q_e - k_1 t$	q_t: amount of adsorbate adsorbed at time (mg/L)<br>q_e: equilibrium adsorption capacity (mg/g)<br>k_1: pesudo-first-order rate constant (1/min)<br>t: time (min)
PSO	$\frac{t}{q_t} = \frac{1}{k_2 \cdot q_e 2} + \frac{t}{q_e}$	k_2: pseudo-second-order-rate constant (L/mg • min)
Intra particle diffusion (ID)	$q_t = k_{id} t^{1/2} + C$	kid: Intraparticle diffusion rate constant ($mg/g \cdot h^{0.5}$)
Langmuir	$\frac{1}{q_e} = \frac{1}{q_m K_L} \frac{1}{C_e} + \frac{1}{q_m}$<br>$R_L = \frac{1}{1 + K_L C_0}$	q_m: maximum adsorption capacity (mg/g)<br>K_L: Langmuir constant (L/mg)<br>C_e: equilibrium adsorbate conceration in solution (mg/L)<br>C_0: initial adsorbate concetration in solution (mg/L)<br>R_L: separation factor
Freundlich	$\ln q_e = \ln K_F + (\frac{1}{n}) \ln C_e$	K_F: Freundlich constant ($mg/g(L/mg)^{1/n}$)<br>n: heterogeneity factor
Temkin	q_e=B $\ln K_T$ + B $\ln C_e$	K_T: Temkin equilibrium binding constant (L/mg)<br>B: Temkin constant (J/mol)
Gibbs Free Energy	$\triangle G^\circ = \triangle H^\circ - T\triangle S^\circ$	$\triangle G^\circ$: Gibbs free energy change (kJ/mol)<br>$\triangle H^\circ$: enthalpy change (J/mol)<br>$\triangle S^\circ$: entropy change (J/mol • K)
